# SMAD3 Host and Tumor Profiling to Identify Locally Advanced Rectal Cancer Patients at High Risk of Poor Response to Neoadjuvant Chemoradiotherapy

**DOI:** 10.3389/fphar.2021.778781

**Published:** 2021-12-24

**Authors:** Elena De Mattia, Vincenzo Canzonieri, Jerry Polesel, Silvia Mezzalira, Chiara Dalle Fratte, Eva Dreussi, Rossana Roncato, Alessia Bignucolo, Roberto Innocente, Claudio Belluco, Salvatore Pucciarelli, Antonino De Paoli, Elisa Palazzari, Giuseppe Toffoli, Erika Cecchin

**Affiliations:** ^1^ Experimental and Clinical Pharmacology, Centro di Riferimento Oncologico di Aviano (CRO) IRCCS, Aviano, Italy; ^2^ Pathology, Centro di Riferimento Oncologico di Aviano (CRO) IRCCS, Aviano, Italy; ^3^ Department of Medical, Surgical and Health Sciences, University of Trieste, Trieste, Italy; ^4^ Unit of Cancer Epidemiology, Centro di Riferimento Oncologico di Aviano (CRO) IRCCS, Aviano, Italy; ^5^ Radiation Oncology, Centro di Riferimento Oncologico di Aviano (CRO) IRCCS, Aviano, Italy; ^6^ Surgical Oncology, Centro di Riferimento Oncologico di Aviano (CRO) IRCCS, Aviano, Italy; ^7^ Clinica Chirurgica II, Padova University, Padova, Italy

**Keywords:** rectal cancer, neoadjuvant chemoradiotherapy, 5-fluorouracil, Smad3, immunohistochemistry, polymorphisms, predictive markers

## Abstract

Identifying patients at risk of poor response to neoadjuvant chemoradiotherapy (nCRT) is an emerging clinical need in locally advanced rectal cancer (LARC). SMAD3 is a key player in the chemoradio-resistance phenotype and its expression is both constitutive and locally induced. The aim was to investigate both host (genetic polymorphisms) and tumor SMAD3 profiling to predict response to nCRT. In a group of 76 LARC patients, SMAD3 and phosphorylated-SMAD3 expression was assessed by immunohistochemistry in preoperative tumor tissue. In an expanded study group (*n* = 378), a set of *SMAD3* polymorphisms (rs35874463, rs1065080, rs1061427, rs17228212, rs744910, and rs745103) was analyzed. Association with tumor regression grade (TRG) and patient prognosis (progression-free survival [PFS] and overall survival [OS]) was assessed. Patients with high tumor expression of SMAD3 had a significantly increased risk of poor response (TRG≥2) [cellularity >55% (OR:10.36, *p* = 0.0004), or moderate/high intensity (OR:5.20, *p* = 0.0038), or an H-score≥1 (OR:9.84, *p* = 0.0004)]. Patients carrying the variant SMAD3 rs745103-G allele had a poorer response (OR:0.48, *p* = 0.0093), a longer OS (HR:0.65, *p* = 0.0307), and a trend for longer PFS (HR:0.75, *p* = 0.0944). Patients who carried both high SMAD3 tumor expression and the wild-type rs745103-A allele had an extremely high risk of not achieving a complete response (OR:13.45, *p* = 0.0005). Host and tumor SMAD3 status might be considered to improve risk stratification of LARC patients to facilitate selection for alternative personalized neoadjuvant strategies including intensified regimens.

## Introduction

A combined modality approach with fluoropyrimidine-based neoadjuvant chemoradiotherapy (nCRT) followed by total mesorectal excision represents the standard of care for patients with locally advanced rectal cancer (LARC) (Yoo and Kim, 2019; Roeder et al., 2020). New treatment trends are based on risk stratification and include regimens with intensified pre-operative chemotherapy, such as total neoadjuvant therapy (TNT), in high-risk cases with poor response and recurrence (Rosello et al., 2018; Fokas et al., 2019; Papaccio et al., 2020; Riesco-Martinez et al., 2020; Bahadoer et al., 2021). The burning clinical question is how to better identify high-risk patients, currently defined only by specific clinical criteria such as clinical T and N stages, distance of tumor from anal verge, involvement of mesorectal fascia, and extramural vascular invasion (Glynne-Jones et al., 2018). Therefore, additional molecular predictors to be integrated in the clinical practice are specifically needed.

SMAD family member 3 (SMAD3) is an attractive candidate for a predictive and prognostic marker in cancer (Moon et al., 2015; Jung et al., 2017). It is a major transcription factor in the transforming growth factor-β (TGF-β) downstream signaling pathway, which is critical for the immunosuppressive and radioresistant phenotype associated with TGF-β (Tauriello and Batlle, 2016; Koveitypour et al., 2019). Within the tumor microenvironment, TGF-β is the most potent suppressor of radiotherapy-triggered anti-tumor T-cell responses (Vanpouille-Box et al., 2015; Wennerberg et al., 2017; Farhood et al., 2020; Liu et al., 2021). Following radiotherapy-mediated activation, TGF-β has been shown to upregulate immunosuppressive T regulatory cells and to downregulate anti-tumor effector cells (i.e., CD8^+^ T lymphocytes and natural killers) (Vanpouille-Box et al., 2015; Wennerberg et al., 2017; Farhood et al., 2020; Liu et al., 2021). The role of SMAD3 in this context is not fully elucidated, although pharmacological approaches targeting SMAD3 have shown some enhancement of the immune response to radiotherapy (Akhurst and Hata, 2012; Rodriguez-Ruiz et al., 2019).

SMAD3 exhibits both constitutive (host-driven) and inducible (tumor-driven) expression in cancer (Jung et al., 2017). Host genetic variations in *SMAD3* can lead to dysregulation of TGF-β signaling. We previously reported a key role of three intronic *SMAD3* germline polymorphisms (rs744910, rs745103, and rs17228212) in predicting response to fluoropyrimidine-based nCRT in LARC patients (Dreussi et al., 2016). On the other hand, SMAD3 expression and activation at the tumor cellular level may serve as a marker for tumor proliferation, metastasis, and patient prognosis. Overexpression of nuclear C-terminal phosphorylated SMAD3 (p-SMAD3) in preoperative tumor samples was indicated to identify LARC patients at higher risk for poor response to fluoropyrimidine-based nCRT (Huang et al., 2015).

This study addresses for the first time both the host and tumor component of SMAD3 profiling through a combined molecular approach. The primary aim of the study was to define the association between the constitutive genetic features of SMAD3, tumor protein expression and their combination, and tumor response to standard nCRT regimens in LARC patients. These findings may improve the upfront identification of high-risk patients who could be proposed for alternative personalized preoperative approaches.

## Materials and Methods

### Study Design and Patient Cohorts

All cases included in the study were selected from a consecutive collection of 617 patients with mid and low (stage II-III) primary adenocarcinoma of the rectum treated with nCRT between March 1994 and November 2015 at Centro di Riferimento Oncologico- IRCCS (CRO) of Aviano, Istituto Oncologico Veneto- IRCCS (IOV) of Padua, and Clinica Chirurgica I of Padua University.

Patients were enrolled in a prospective study protocol with the aim of revealing predictive and prognostic molecular biomarkers. The inclusion criteria were as follows: 1) Histologically confirmed diagnosis of primary resectable LARC; 2) confirmed absence of distant metastases; 3) age ≥18 years; 4) self-reported Caucasian ethnicity; 5) disease stage T3-T4 and N0-N2; 6) performance status (World Health Organization) 0–2; 7) normal bone marrow, renal, and liver function.

**FIGURE 1:**
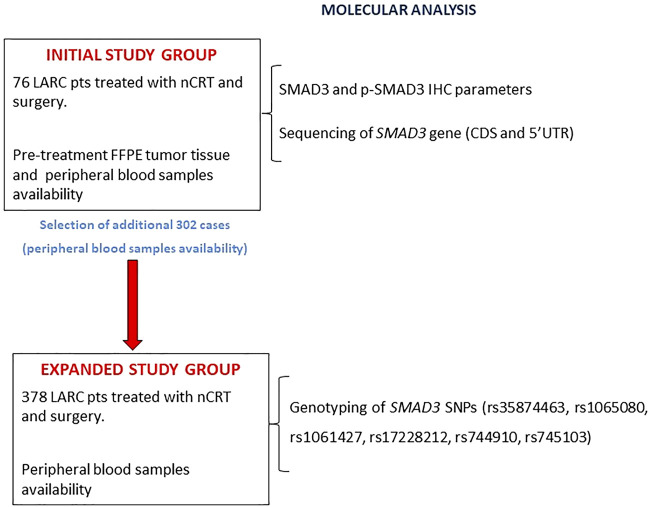
Flowchart of the study design. Abbreviations: CDS, coding sequence; FFPE, formalin-fixed, paraffin-embedded; IHC, immunohistochemistry; LARC, locally advanced rectal cancer; nCRT, neoadjuvant chemoradiotherapy; pts, patients; p-SMAD3, phosphorylated SMAD3; SNP, single nucleotide polymorphism.

The study design (Figure 1) included an initial study group of 76 patients with both a formalin-fixed paraffin-embedded (FFPE) pre-treatment tumor biopsy and a peripheral blood sample. In this group, SMAD3 and *p*-SMAD3 expressions were determined by immunohistochemistry (IHC) and tested for association with tumor regression grade (TRG). The *SMAD3* gene was sequenced on germline DNA extracted from peripheral blood.

The initial study group was then expanded by adding 302 patients with an available peripheral blood sample. In the expanded study group (*n* = 378), the impact of a set of *SMAD3* genetic polymorphisms (rs35874463, rs1065080, rs1061427, rs17228212, rs744910, and rs745103) on TRG and patient prognosis (progression-free survival [PFS] and overall survival [OS]) were investigated.

The study protocol conformed to the ethical guidelines of the 1975 Declaration of Helsinki. The protocol was approved by the Ethical Committee of all participating institutions, and all patients gave written informed consent for research purposes before participating in the study. All experiments were conducted in accordance with the relevant guidelines and regulations of the Centro di Riferimento Oncologico di Aviano.

### Tumor Treatment, Response Evaluation and Follow-Up

All patients received a nCRT based on fluoropyrimidines (either 5-fluorouracil [5-FU] or capecitabine) concomitant with radiotherapy as previously described (Cecchin et al., 2020). A radiation dose of 50.4 Gy, administered in 28 daily fractions over a period of 5.5 weeks, was given as standard treatment in most cases. A subset of patients received nCRT intensification either by a concomitant radiotherapy boost to the bulky tumor (cumulative radiation dose of 55.0 Gy administered by a concomitant boost of 1Gy 2 times/week for 5 weeks) or by concomitant administration of oxaliplatin according to enrollment in an institutional randomized clinical trial (Valentini et al., 2019). Six to eight weeks after completion of the chemoradiotherapy program, patients underwent either Total Mesorectal Excision or local excision, depending on clinical response to treatment; adjuvant chemotherapy was optional depending on pathologic stage after surgery.

Pathological tumor staging of the resected specimens was performed according to the American Joint Committee on Cancer TNM classification guidelines (AJCC American Joint Committee on Cancer, 1997). The whole residual tumoral area was sampled for histopathological examination and ypT evaluation, as well as assessment of mesorectal surgical margin status and lymph nodal changes. Pathological response to nCRT of the primary tumor was recorded according to the TRG criteria proposed by Mandard (Mandard et al., 1994). Survival and tumor progression data were obtained by active follow-up.

### SMAD3 and p-SMAD3 Protein Expression

**FIGURE 2:**
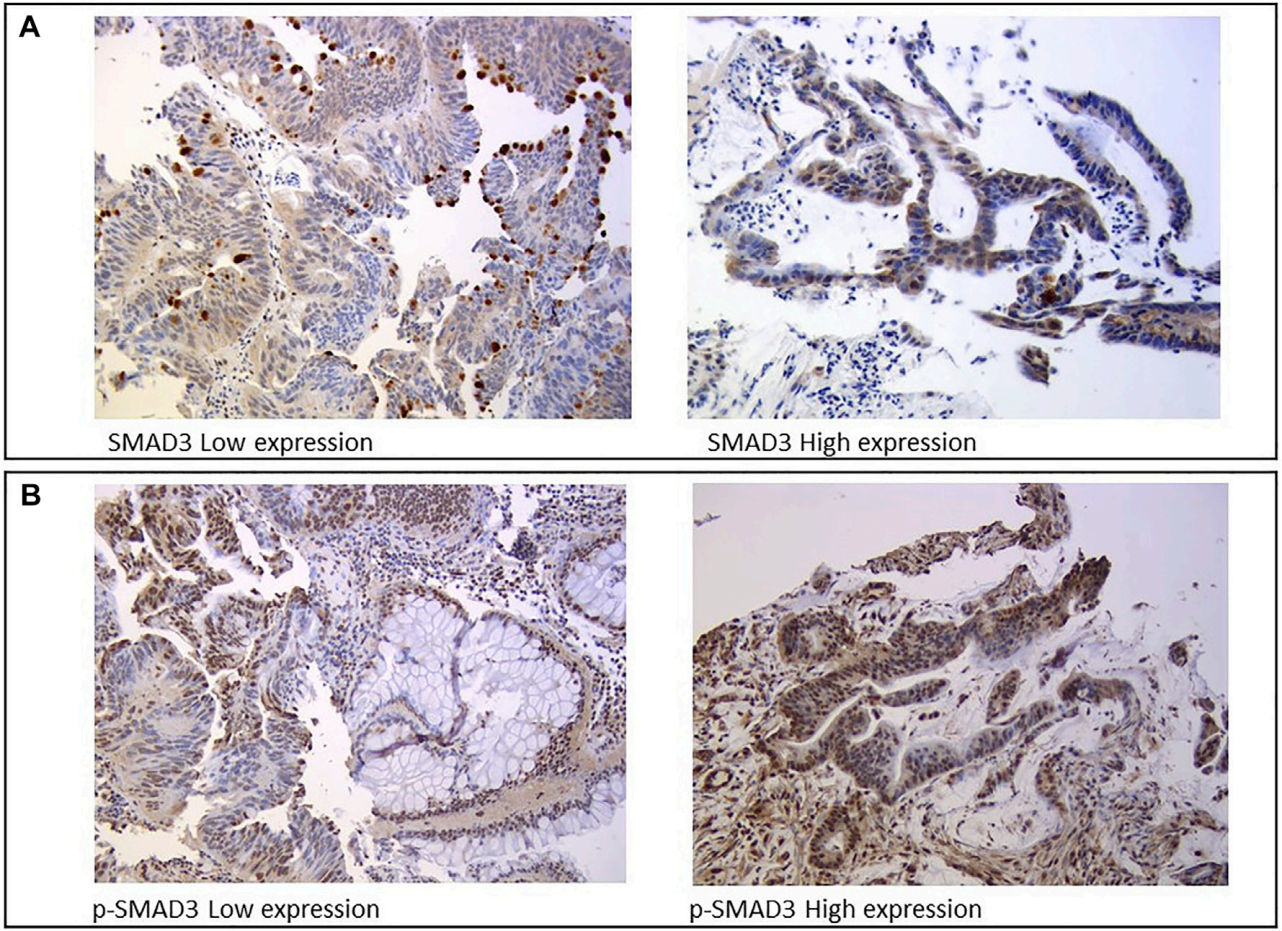
Immunohistochemistry staining of **(A)** SMAD3 and **(B)** and its phosphorylated form (p-SMAD3).

Protein expression was assessed by IHC analysis on FFPE samples from tumor biopsies collected during staging colonoscopy prior to nCRT. Three µm-thick sections were serially cut from each FFPE block, one slide was stained with hematoxylin and eosin, and the remaining slides were used for IHC analysis of SMAD3 and p-SMAD3, which were independently reviewed and scored by two trained pathologists blinded to patient clinical information. SMAD3 expression was assessed in the apical part of the cytoplasm using the mouse SMAD3 monoclonal antibody (M01, clone 2C12, and Abnova) (Figure 2A). The nuclear phosphorylated form of SMAD3 was evaluated using the rabbit SMAD3 polyclonal antibody (phospho S423/425, Abnova) (Figure 2B).

SMAD3 and p-SMAD3 protein expressions were assessed based on IHC staining intensity (0, absent; 1, weak; 3, moderate; 4, strong) and cellularity (from 0 to 100%), which was defined as the percentage of positively stained tumor cells on the total number of visible tumor cells. Staining intensity and cellularity were then combined into an H-score ranging from 0 to 3, as previously described (Huang et al., 2015).

### *SMAD3* Sequencing

Genomic DNA was extracted from peripheral blood samples using the automated extractor BioRobot EZ1 (“EZ1 DNA Blood Kit 350 μl” kit; Qiagen).

Sequencing of the *SMAD3* gene (ENSG00000166949; reference transcript ENST00000327367.9) was performed using the Sanger method. The assay was designed to map the coding region of the nine exons, their adjacent splice junctions (20 bases upstream and downstream of the exon), and the 5′-untranslated region (approximately 70 bases upstream the start codon AUG). PCR primers were selected using Primer3Plus (http://www.bioinformatics.nl/cgi-bin/primer3plus/primer3plus.cgi). PCR reactions were performed in an Eppendorf Mastercycler gradient, using TaqGold DNA Polymerase (ThermoFisher-Applied Biosystems). Purified reactions were sequenced using the Big Dye Terminator kit (ThermoFisher-Applied Biosystems) on an ABI PRISM 3130 capillary sequencer. Both reverse and forward primers were used to sequence the target regions. Chromatograms were visualized using Chromas software version 2.5 and aligned to the human *SMAD3* reference genome sequence through the T-Coffee Multiple Sequence Alignment Server (http://tcoffee.crg.cat/) to identify genetic variants.

### *SMAD3* Polymorphisms Genotyping

Six polymorphisms (rs35874463, rs1065080, rs1061427, rs17228212, rs744910, and rs745103) were tested in the expanded study group using a predesigned TaqMan SNP genotyping assay based on the allelic discrimination method using fluorescent probes. All commercial TaqMan assays were purchased from ThermoFisher-Applied Biosystems (https://www.thermofisher.com) and analyses were performed on an ABI 7500 instrument (ThermoFisher-Applied Biosystems) according to the manufacturer’s instructions. Positive and negative control samples were included in each analysis. Further details of the analytical procedures are available on request.

### Bioinformatic Analysis

Functional prediction of the putative effect of candidate polymorphisms was performed using three online software: HaploReg v4.1 (https://pubs.broadinstitute.org/mammals/haploreg/haploreg.php), RegulomeDB v2.0 (https://regulomedb.org/regulome-search/) and Ensembl’s Variant Effect Predictor (VEP) Ensembl release 103, February 2021 (https://www.ensembl.org/info/docs/tools/vep/index.html). For haploblock identification by HaploReg, a stringency of r^2^ = 0.80 and a 1,000 Genomes Project (EUR) dataset were chosen.

### Statistical Analysis

The association between IHC parameters and TRG was investigated in the initial study group: each parameter was dichotomized according to the optimal cut-off from a receiver operating characteristic (ROC) curve discriminating TRG1 from TRG2-4 patients. Odds ratio (OR) for TRG2-4 and corresponding confidence intervals (CI) were calculated using the unconditional logistic regression model, adjusting for sex, age, distance from anal verge, total RT dose, time between RT and surgery, and oxaliplatin use.

The association between *SMAD3* polymorphisms and oncological endpoints was investigated in the expanded study group. Hardy-Weinberg equilibrium was first tested by a permutation procedure based on an exact test. OR for TRG2-4 and the corresponding CI were calculated by an unconditional logistic regression model, considering dominant, recessive, and additive genetic models by combining heterozygous with homozygous genotypes; the best-fitting genetic model was selected according to the Wald chi-square test. Furthermore, the association between *SMAD3* polymorphisms and PFS/OS was evaluated by survival analysis. For each patient, the risk time was calculated from the date of surgery to the date of the event of interest (i.e., progression or death for PFS and death for OS) or the last follow-up, whichever occurred first. The hazard ratio (HR) and corresponding CI were calculated through Cox proportional hazard model, adjusting for study, sex, age, distance from anal verge, total RT dose, time between RT and surgery, and use of oxaliplatin. Statistical significance was claimed for *p* < 0.05 (two-tailed).

## Results

### SMAD3 and p-SMAD3 Tumor Expression and Tumor Response (TRG)

The main demographic, clinical and pathological characteristics of the initial study group (*n* = 76) are summarized in Table 1.

**TABLE 1:** Socio-demographic and clinical characteristic of locally advanced rectal cancer patients enrolled in the study.

	Initial study group (*n* = 76)		Expanded study group (*n* = 378)	
	n	(%)	n	(%)
Sex				
Female	28	(36.8)	117	(30.9)
Male	48	(63.2)	261	(69.1)
Age, years (median, range)	62	(24-81)	63	(20-87)
Tumor distance from anal margin (cm)				
<8	54	(71.1)	260	(68.8)
≥8	22	(29.0)	118	(31.2)
Total dose of radiation therapy (Gy)				
50.4	49	(64.5)	278	(73.5)
55.0	27	(35.5)	80	(21.2)
Unknown	0	(0.0)	20	(5.3)
Surgical procedures				
Low anterior resection	42	(55.3)	231	(61.1)
Abdominal perineal resection	9	(11.8)	43	(11.4)
Local excision	17	(22.4)	41	(10.8)
Hartmann’s	2	(2.6)	10	(2.6)
Colo-anal anastomosis	0	(0.0)	27	(7.1)
Other	6	(7.9)	23	(6.1)
Unknown	0	(0.0)	3	(0.8)
Preoperative Chemotherapy				
Fluoropyrimidines				
5-Fluorouracil	4	(5.3)	131	(34.7)
Capecitabine	65	(85.5)	205	(54.2)
Unknown	7	(9.2)	42	(11.1)
Association therapy with oxaliplatin				
*No*	54	(71.1)	284	(75.1)
*Yes*	22	(29.0)	94	(24.9)
Adjuvant therapy				
Yes	39	(51.3)	191	(50.5)
No	34	(44.7)	162	(42.9)
Unknown	3	(4.0)	25	(6.6)
Tumor Regression grade				
1	24	(31.6)	100	(26.5)
2	11	(14.5)	68	(18.0)
3	36	(47.4)	133	(35.2)
4	5	(6.6)	64	(16.9)
5	0	(0.0)	13	(3.4)

SMAD3 parameters were significantly associated with TRG (Table 2). SMAD3 cellularity above 55% or moderate/high immunostaining intensity were both associated with higher risk of TRG2-4 (OR = 10.36 CI:2.81-38.18, and OR = 5.20 CI:1.70-15.88, respectively). These results were confirmed when H-score (cut-off = 1) (OR = 9.84 CI:2.75-34.40) was taken into account. The association with tumor response was not significant for pSMAD3 cellularity (cut-off = 85%) or immunostaining intensity, while it became significant when H-score (cut-off = 2) was considered (OR = 4.23 CI:1.31-13.64) (Table 2).

**TABLE 2:** Association between immunohistochemistry (IHC) parameters and tumor regression grade (TRG), in the initial study group (*n* = 76). Associations with P-value <0.05 are in bold.

IHC parameters^a^	TRG1		TRG2-4		OR (95% CI)^b^
	n	(%)	n	(%)	
SMAD3 cellularity					
≤55%	19	(50.0)	19	(50.0)	**Reference**
>55%	5	(13.2)	33	(86.8)	**10.36 (2.81-38.18)**
			***p* = 0.0011** **^c^**		***p* = 0.0004** **^d^**
SMAD3 intensity					
Low	14	(51.9)	13	(48.1)	**Reference**
Moderate/High	10	(20.4)	39	(79.6)	**5.20 (1.70-15.88)**
			***p* = 0.0090** **^c^**		***p* = 0.0038** **^d^**
SMAD3 score (Huang^f^)					
0	14	(60.9)	9	(39.1)	**Reference**
≥1	10	(18.9)	43	(81.1)	**9.84 (2.75-34.40)**
			***p* = 0.0009** **^c^**		***p* = 0.0004** **^d^**
p-SMAD3 cellularity					
<85%	19	(34.5)	36	(65.5)	Reference
≥85%	5	(23.8)	16	(76.2)	1.84 (0.54-6.31)
			*p* = 0.4212^c^		*p* = 0.3304^d^
p-SMAD3 intensity					
Low-Moderate	12	(41.4)	17	(58.6)	Reference
High	12	(25.5)	35	(74.5)	2.56 (0.85-7.76)
			*p* = 0.2046^c^		*p* = 0.0957^d^
p-SMAD3 score (Huang^f^)					
≤2	17	(44.7)	21	(55.3)	**Reference**
>2	7	(18.4)	31	(81.6)	**4.23 (1.31-13.64)**
			***p* = 0.0253** **^c^**		***p* = 0.0158** **^d^**

Abbreviations: 95% CI, 95% confidence interval; OR, odds ratio; p-SMAD3, phosphorylated SMAD3.

a Optimal cut-off was calculated by ROC analysis.

b Estimated using an unconditional logistic regression model adjusting for sex, age (<60, 60-69, and ≥70 years), distance from anal verge (<5, 5-6, and ≥7 cm), total radiotherapy dose (<55.0, 55.0 Gy), time between radiotherapy and surgery (<60, ≥60 days), and oxaliplatin use (no, yes).

c Fisher’s exact test.

d Wald χ2 test.

f “0“, complete absence of staining; “1” weak staining in more than 50% of positive cells or with moderate staining in less than 50% of positive cells; “2”, moderate positive staining in more than 50% of cells, or with strong staining in less than 50 %t of cells; “3”, strong staining in more than 50% of cells (according to Huang et al., 2015).

A similar association trend, though not significant, was observed when focusing on the subgroup of patients (*n* = 22) treated with a combination chemotherapy including fluoropyrimidines and oxaliplatin (data not shown).

### *SMAD3* Genetic Sequencing

A total of 1,619bp in the *SMAD3* gene was sequenced by Sanger method. Four genetic variants (2.47 variants/kbp) (i.e., rs35874463, rs1065080, rs117185005, and rs1061427) were detected in 76 evaluable patients (Supplementary Figure S1). Their main characteristics and genotype frequencies are listed in Table 3 and are consistent with the 1,000 Genomes Project data for the European population. Further details have been reported below in the results of the bioinformatic analysis.

**TABLE 3:** The main features and genotype frequency of the identified *SMAD3* polymorphisms.

Rs ID	Genomic position (GRCh38)	Nucleotide change	Typology	Location	Amino acid change	MAF, EUR/TSI^a^	Genotypes frequency in study group (*n* = 76)			
							AA	Aa	aa	MAF
rs1065080	chr15:67164997	CT **A**/CT **G**	synonymous	Exon 2	Leu103Leu	A: 0.139/0.103	GG: 0.800 (60)	GA: 0.173 (13)	AA: 0.027 (2)	A: 0.113
rs35874463	chr15:67165360	ATC/GTC	missense	Exon 3	Ile170Val	G: 0.053/0.037	AA: 0.920 (69)	AG: 0.080 (6)	GG: 0	G: 0.04
rs117185005	chr15:67181452	AT **C**/AT **T**	synonymous	Exon 6	Ile290Ile	T: 0.024/0.014	CC: 0.973 (72)	CT: 0.027 (2)	TT: (0)	T: 0.014
rs1061427	chr15:67066140	CCGC **G** CG/CCGC **A** CG	5′ regulatory region	5′UTR	—	A: 0.246/0.206	GG: 0.635 (47)	GA: 0.257 (19)	AA: 0.108 (8)	A: 0.236

Abbreviations: MAF, minor allele frequency.

a 1000 Genomes Project Phase 3 (32), European (EUR) and Toscany in Italy (TSI) population.

### *SMAD3* Polymorphisms and Tumor Response and Prognosis

The main demographic, clinical and pathological characteristics of the expanded study population (*n* = 378) are listed in Table 1.

Three variants identified by Sanger sequencing (i.e., rs35874463, rs1065080, and rs1061427) and the three intronic variants (i.e., rs17228212, rs744910, and rs745103) studied in our previous work (Dreussi et al., 2016) (Supplementary Figure S1) were tested. The rs117185005 detected by sequencing was not analyzed in the expanded study group due to its very low frequency.

The predictive effect we previously observed for rs744910 and rs745103 on TRG (Dreussi et al., 2016) was validated (Table 4). Patients carrying the rs744910-GG (*p* = 0.0176) or rs745103-GG (*p* = 0.0093) genotype had a significantly reduced risk of poor response (TRG≥2). Only a trend was observed for rs17228212, with the C-allele being associated with an increased risk of getting TRG2-5 (*p* = 0.0848), consistent with our previous results (Dreussi et al., 2016).

**TABLE 4:** (A) Odds ratio (OR) and 95% confidence interval (CI) for tumor regression grade (TRG) and (B) Hazard ratios (HR) and 95% CI for progression-free survival (PFS) and overall survival (OS) in the expanded study group (*n* = 378) according to gene polymorphisms (SNP). Associations with *P*-value < 0.05 are in bold.

(A)										
SNP	Base change	Genotype frequency						Genetic model	OR (95% CI)^a^	*p*-value
		TRG1			TRG2-5					
		AA	Aa	Aa	AA	Aa	aa			
rs17228212	T > C	0.632	0.327	0.040	0.544	0.365	0.091	Additive	1.42 (0.95–2.12)	0.0848
rs744910	A > G	0.202	0.475	0.323	0.256	0.542	0.202	Recessive	0.52 (0.31–0.89)	**0.0176**
rs745103	A > G	0.222	0.475	0.303	0.272	0.544	0.184	Recessive	0.48 (0.28–0.83)	**0.0093**
rs1065080	G > A	0.765	0.214	0.020	0.803	0.182	0.015	Recessive	0.71 (0.12–4.12)	0.7009
rs35874463	A > G	0.969	0.031	0.000	0.919	0.078	0.004	Dominant	2.74 (0.79–9.52)	0.1138
rs1061427	G > A	0.563	0.354	0.083	0.629	0.309	0.062	Additive	0.79 (0.54–1.16)	0.2267

(B)							
SNPs	Base change	PFS			OS		
		Genetic model	HR (95% CI)^a^	*p*-value	Genetic model	HR (95% CI)^a^	*p*-value
rs17228212	T > C	Recessive	0.75 (0.30–1.88)	0.5365	Recessive	0.57 (0.18–1.84)	0.3486
rs744910	A > G	Recessive	0.66 (0.37–1.18)	0.1614	Recessive	0.86 (0.45–1.45)	0.6536
rs745103	A > G	Additive	0.75 (0.54–1.05)	0.0944	Additive	0.65 (0.44–0.96)	**0.0307**
rs1065080	G > A	Dominant	1.19 (0.71–2.01)	0.5092	Additive	1.28 (0.74–2.19)	0.3762
rs35874463	A > G	Dominant	1.00 (0.39–2.51)	0.9919	Dominant	0.99 (0.35–2.79)	0.9838
rs1061427	G > A	Additive	0.70 (0.47–1.03)	0.0693	Additive	0.71 (0.45–1.12)	0.1418

a Estimated from Cox proportional hazards model, adjusted for sex, age (<60, 60-69, and ≥70 years), distance from anal verge (<5, 5-6, and ≥7 cm), total radiotherapy dose (<55.0, 55.0 Gy), time between radiotherapy and surgery (<60, ≥60 days), and oxaliplatin use (no, yes).

During a median follow-up of 56 months, 62 patients (16.4%) died, while 89 patients (23.5%) had disease recurrence. The five-years OS was 84.8% (80.2–88.4%), while the 5-years PFS was 74.8% (69.6–79.3%).

**FIGURE 3:**
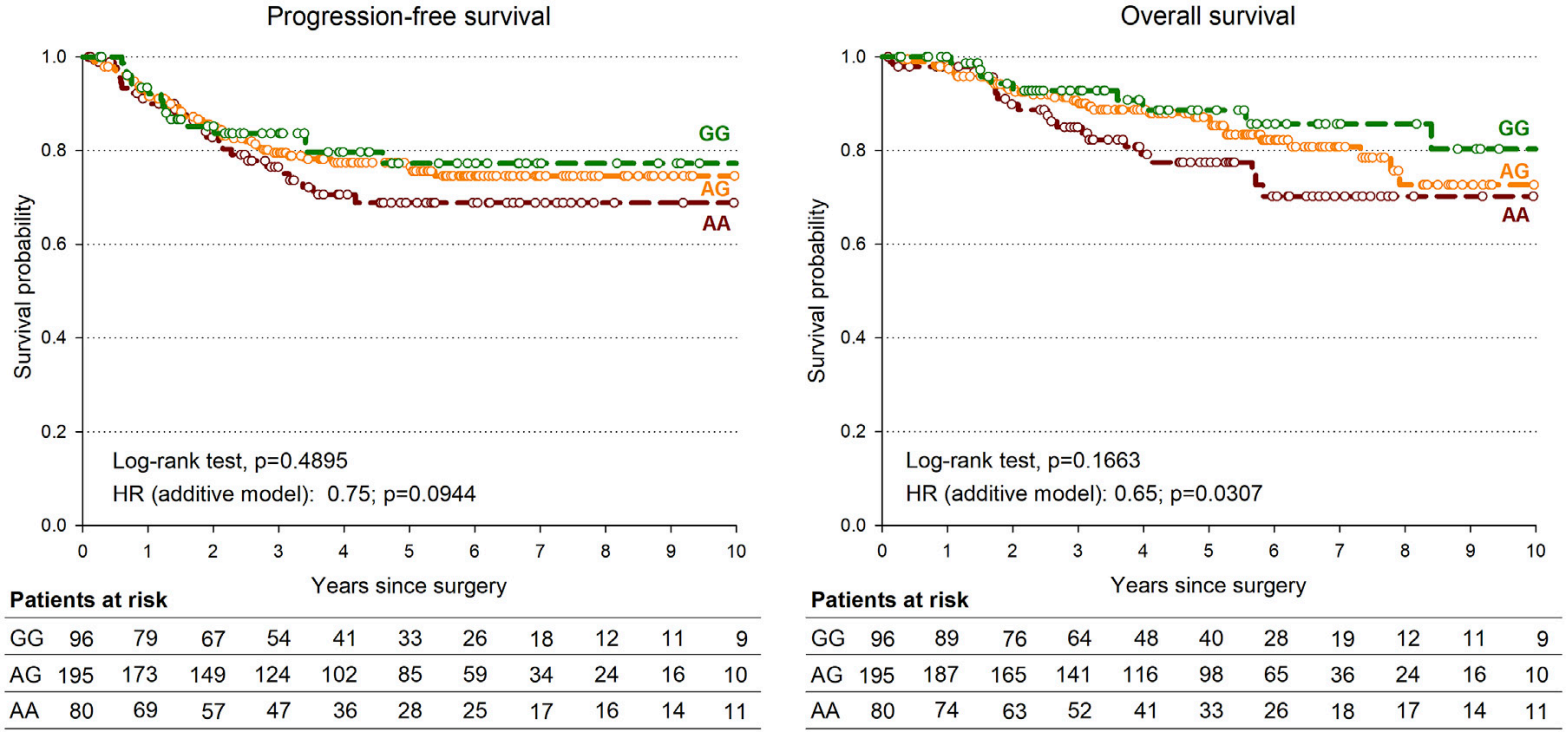
Kaplan-Meier estimates of overall survival **(A)** and progression-free survival **(B)** according to *SMAD3*-rs745103 polymorphism in the expanded study group (*n* = 378).

Only the rs745103 variant was significantly associated with patient survival (Table 4), with the G-allele associated with longer OS (*p* = 0.0307). A non-significant association trend was observed for longer PFS (*p* = 0.0944) (Table 4). The log-rank test was not significant, but the Kaplan-Meier curves presented in Figure 3 showed a trend consistent with the results of the Cox analysis. The effect of rs745103 on OS and PFS was consistent with that observed for tumor response to treatment.

### Combined Predictive Effect of SMAD3 Tumor Expression, *SMAD3*-rs745103 and *SMAD3*-rs744910, on Tumor Response

Patients who were part of the initial study group characterized for both SMAD3 tumor expression and *SMAD3*-rs745103 and rs744910 genotypes (74/76 patients) were considered for the combined analysis.

We started splitting patients into high- and low-risk groups incomplete response based on tumor IHC expression parameters (Table 5). We then performed a stepwise forward regression analysis by including patient’s genotype for *SMAD3*-rs744910 and *SMAD3*-rs745103 in the model. Patients carrying both the IHC and genetic unfavorable features were considered at “high-risk” and compared with the others. Inclusion of *SMAD3*-rs745103 information in the IHC score improved identification of patients at increased risk (i.e., OR) of not completely responding to treatment (TRG2-4) compared to using only IHC features. The addition of *SMAD3*-rs744910 information did not further improve the model’s capacity to identify patients at high-risk of poor response.

**TABLE 5:** Combined predictive effect of SMAD3 tumor expression, SMAD3-rs745103 and SMAD3-rs744910 on tumor response. Associations with *P*-value < 0.05 are in bold.

IHC parameters	Risk	Expression Level^a^ (*n* = 76)	OR (95% CI)^b^	Expression Level^a^ and rs745103 (*n* = 74)	OR (95% CI)^b^	Expression Level^a^, rs745103, and rs744910 (*n* = 74)	OR (95% CI)^b^
SMAD3 cellularity	Low	≤55%	**Reference**	≤55% or GG	**Reference**	≤55% or GG or GG	**Reference**
	High	>55%	**10.36 (2.81-38.18)**	>55% and AA/AG	**13.45 (3.14-57.57)**	>55% and AA/AG and AA/AG	**10.15 (2.39-43.19)**
			***p* = 0.0004** **^c^**		***p* = 0.0005** **^c^**		***p* = 0.0017** **^c^**
SMAD3 intensity	Low	Low	**Reference**	Low or GG	**Reference**	Low or GG or GG	**Reference**
	High	Moderate/High	**5.20 (1.70-15.88)**	Moderate/High and AA/AG	**8.17 (2.35-28.40)**	Moderate/High and AA/AG and AA/AG	**5.83 (1.74-19.53)**
			***p* = 0.0038** **^c^**		***p* = 0.0010** **^c^**		***p* = 0.0043** **^c^**
SMAD3 score (Huang)^d^	Low	0	**Reference**	0 or GG	**Reference**	0 or GG or GG	**Reference**
	High	≥1	**9.84 (2.75-34.40)**	≥1 and AA/AG	**11.41 (3.19-40.79)**	≥1 and AA/AG and AA/AG	**6.85 (2.07-22.67)**
			***p* = 0.0004** **^c^**		***p* = 0.0002** **^c^**		***p* = 0.0016** **^c^**
p-SMAD3 cellularity		<85%	Reference	<85% or GG	Reference	<85% or GG or GG	Reference
		≥85%	1.84 (0.54-6.31)	≥85% and AA/AG	1.98 (0.52-7.56)	≥85% and AA/AG and AA/AG	1.53 (0.39-5.97)
			*p* = 0.3304^c^		*p* = 0.3195^c^		*p* = 0.5370^c^
p-SMAD3 intensity	Low	Low-Moderate	Reference	Low-Moderate or GG	Reference	Low-Moderate or GG or GG	Reference
	High	High	2.56 (0.85-7.76)	High and AA/AG	2.87 (0.90-9.09)	High and AA/AG and AA/AG	2.06 (0.68-6.28)
			*p* = 0.0957^c^		*p* = 0.0739^c^		*p* = 0.2033^c^
p-SMAD3 score (Huang)^d^	Low	≤2	**Reference**	≤2 or GG	**Reference**	≤2 or GG or GG	Reference
	High	>2	**4.23 (1.31-13.64)**	>2 and AA/AG	**4.90 (1.27-18.99)**	>2 and AA/AG and AA/AG	3.39 (0.92-12.51)
			***p* = 0.0158** **^c^**		***p* = 0.0214** **^c^**		*p* = 0.0670^c^

Abbreviations: 95% CI, 95% confidence interval; OR, odds ratio; p-SMAD3, phosphorylated SMAD3.

a Optimal cut-off was calculated by ROC analysis.

b ORs, for TRG2-4 vs TRG1 were estimated from an unconditional logistic regression model adjusting for sex, age (<60, 60-69, and ≥70 years), distance from anal verge (<5, 5-6, and ≥7 cm), total radiotherapy dose (<55.0, 55.0 Gy), time between radiotherapy and surgery (<60, ≥60 days), and oxaliplatin use (no, yes).

c Wald χ2 test.

d “0”, complete absence of staining; “1” weak staining in more than 50% of positive cells or with moderate staining in less than 50% of positive cells; “2”, moderate positive staining in more than 50% of cells, or with strong staining in less than 50 %t of cells; “3”, strong staining in more than 50% of cells (according to Huang et al., 2015).

### Results of the Bioinformatic Analysis and Association of polymorphisms With Tumor Expression

The *in silico* prediction of the possible functional effect of the four genetic variants identified by Sanger sequencing is shown in Supplementary Table S1A. One missense polymorphism was detected by Sanger Sequencing, the rs35874463 (Ile170Val, and exon 3). This variant is located in the linker region of SMAD3, that is required for TGFbeta-mediated transcriptional activity and acts synergistically with the MH2 domain. Despite its location in a critical region, all *in silico* tools predicted that rs35874463 has a tolerated or benign im-pact on protein functionality. However, an impact of this variant on SMAD3 expression through alteration of transcriptional regulation or epigenetic control could not be ruled out (RegulomeDb score of 2b). Two synonymous polymorphisms were found, the rs1065080 (Leu103Leu, exon 2), and rs117185005 (Ile290Ile, exon 6). Rs1065080 is located in the MH1 domain required for DNA binding; MH1 domain also binds zinc ions, which are necessary for its function. Rs1065080 was predicted to have a regulatory effect by potentially altering the binding site for some transcriptional regulators including the CCCTC-binding factor (CTCF). Rs117185005 is located in the MSH2 domain, which is required for both homomeric and heteromeric interactions, and transcriptional regulation and nuclear import. This polymorphism, situated one base upstream of the end of exon 6, may alter the splicing pattern of the gene. Rs117185005 was also found to change the consensus motif for the CRCF regulator. A variant was identified in the 5′UTR region, the rs1061427. This polymorphism is thought to have a moderate effect on gene expression by broadly altering regulatory chromatin states and the consensus motif for transcriptional factors.

A summary of the available in silico functional data for the *SMAD3* intronic variants (rs745103, rs744910, and rs17228212) is presented in Supplementary Table S1B. The rs745103 polymorphism could have a moderate impact on gene functionality and/or expression, as it broadly alters regulatory chromatin states (i.e., 3 promoter histone marks, 21 enhancer histone marks, 16 DNAse items), proteins bound (i.e., 1 hit) and motifs (i.e., 4 motifs changed), according to the prediction of the HaploReg tool. This effect was summarized by a RegulomeDB rank score equal to 4 (i.e., transcription factors binding + DNase peak data) and a probability score equal to 0.60906. The VEP tool showed a CADD score of 0.780 and a conservation GERPP score of −3.18. HaploReg detected no other polymorphisms in the *SMAD3*-rs745103 haploblock (r^2^ > 0.8). Similar results were obtained for rs744910 and rs17228212. Rs744910 could potentially affect chromatin architecture and DNA methylation pattern (10 enhancer histone marks), and ultimately DNA accessibility for gene transcription. This variant also resulted in DNAse hypersensitivity (7 DNAse items) and is located in a transcriptional binding element (2 altered motifs) with a result-ing impact on the regulation of protein expression (NHGRI-EBI GWAS and eQTL hits). These effects were globally summarized with a RegulomeDB rank score equal to 3a (i.e., transcription factors binding, + any motif + DNase peak data) and a probability score equal to 0.85505. The VEP tool indicated a CADD score of 5.153 and a conservation GERPP score of 0.22. Rs17228212 was predicted to have an impact on *SMAD3* gene functionality and/or expression by potentially altering the chromatin architecture, nucleosomal positioning, and DNA methylation pattern (i.e., 2 promoter histone marks, 15 enhancer histone marks, and 6 DNAse items). Furthermore, this polymorphism is located in a transcriptional binding element (2 altered motifs) with a consequent effect on protein ex-pression (NHGRI-EBI GWAS and eQTL hits). RegulomeDB provided a rank score equal to 3a (i.e., transcription factors binding, + matched transcription factors motif + matched DNase Footprint + DNase peak) and a probability score equal to 0.47489. The VEP tool indicated a CADD score of 4.384 and a conservation GERPP score of -3.02. Use of HaploReg revealed that 2 and 10 additional genetic variants are tagged by *SMAD3*-rs744910 and rs17228212 (r2>0.8) respectively.

The association between these seven polymorphisms and the baseline expression level of SMAD3 in tumor tissue was tested, but no statistically significant association was found (data not shown).

## Discussion

Selection for innovative intensified nCRT programs, such as TNT, of LARC patients at high risk for poor clinical outcome is currently based solely on clinical parameters. The integration of new predictive markers could improve existing clinical risk algorithms to achieve a precision medicine approach.

Our most important finding was the identification of some host *SMAD3* genetic polymorphisms (rs744910, rs745103) and SMAD3 protein expression in pre-treatment tumor tissue as predictive markers of response to neoadjuvant treatment in LARC. For the first time, we demonstrated that the combination of SMAD3 tumor expression level with host *SMAD3*-rs745103 genotype could identify smaller groups of patients at significantly higher risk of not responding to nCRT treatment compared to individual molecular parameters. This preliminary result highlights the advantage of integrating multiple molecular markers (host- and tumor-related) for predicting the likelihood of response to treatment. It also suggests that they could independently account for the constitutive (host) and inducible (tumor) SMAD3 effect on the treatment outcome.

SMAD3 is a key transcription factor in the TGF-β signaling pathway and could contribute to determine the immunosuppressive phenotype associated with TGF-β activation and to counteract the ability of radiotherapy to induce an effective antitumor immune response (Vanpouille-Box et al., 2015; Tauriello and Batlle, 2016; Wennerberg et al., 2017; Farhood et al., 2020; Liu et al., 2021). SMAD3 could also reduce DNA damage response and promote cell survival, invasion, migration, and epithelial-mesenchymal transition (Choi et al., 2016; Lee et al., 2016; Li et al., 2017; Jiang et al., 2019; Niu et al., 2020). In the present work, high SMAD3 expression was associated with poorer response to nCRT. Accordingly, recent *in vitro* studies have reported that silencing of SMAD3 resulted in increased sensitivity to radiotherapy (Jiang et al., 2019; Niu et al., 2020) and that higher SMAD3 expression is associated with shorter survival and higher risk of recurrence after radiotherapy (Niu et al., 2020). Huang and colleagues (Huang et al., 2015), reported that high preoperative p-SMAD3 tumor expression could be a potential predictor of poor response to nCRT in LARC patients.

*SMAD3*-rs745103 and rs744910 proved to be a predictive marker of poor response to treatment, confirming in this larger and prospective population our previous pharmacogenetic analyses (Dreussi et al., 2016). Furthermore, a prognostic impact of *SMAD3*-rs745103 on OS and PFS was highlighted. It could be hypothesized that the genetic variant might affect the constitutive expression/activity of SMAD3, which in turn modifies the TGF-β-related transcriptional response and influences the antitumor efficacy of nCRT. *SMAD3*-rs745103 is an intronic variant and our bioinformatic prediction analysis indicated that it could moderately affect gene functionality and/or expression.

Beyond the effect on radiotherapy, it should be noted that the TGF-β/SMAD3 pathway was also reported to be involved in the mechanism of resistance to chemotherapeutics, including 5-FU, in colorectal cancer by modulating TGF-β downstream effectors with pro-proliferative, and pro-metastatic and anti-apoptotic effects (Moon et al., 2015; Romano et al., 2016). On the other hand, suppression of the TGF-β/SMAD3 cascade was shown to inhibit 5-FU-induced gene transcription and restore the sensitivity of 5-FU chemoresistant cells (Romano et al., 2016).

Based on our gene sequencing results, only four variants were identified, corresponding to a variation rate of 2.47 variants/kbp. This is significantly lower compared to the ExAc project data (1 variant/8bp within exome intervals) (Lek et al., 2016). All four variants were predicted by *in silico* analysis to have minimal impact on SMAD3 functionality and/or expression. *SMAD3* was found to be a highly conserved gene, consistent with its basic biological role, and including regulation of immune response.

The study was not devoid of limitations. First, it was performed on a large series of prospectively enrolled LARC patients, diagnostic biopsy was only available for a subset to allow an integrated molecular approach focusing on both host and tumor. In any case, the rarity of the pathology and the novelty of the results warrant attention, although further validation in independent groups of patients is needed. Second, even if preliminary *in silico* results support a possible phenotypic impact, the precise functional significance of *SMAD3*-rs745103 is still unknown, and confirmatory functional analyses are required. Third, considering the effect of SMAD3 on the tumor aggressiveness phenotype, regardless the impact on chemoradiotherapy (Liu et al., 2015; Romano et al., 2016; Tauriello and Batlle, 2016), a non-treated control group would have been helpful to clarify the contribution of SMAD3 on tumor response to treatment.

This study demonstrates that tumor SMAD3 protein expression and germline genotype could predict response to fluoropyrimidine-based nCRT. Its findings suggest the relevance of the TGF-β/SMAD3 pathway in determining the success of nCRT and may help elucidate the molecular mechanisms underlying response to chemoradiotherapy. The crucial role of TGFβ in determining the sensitivity to radiation therapy is well-recognized and the inhibition of TGFβ signaling by emerging pharmacological interventions (i.e., receptor kinase inhibitors, TGFβ-directed monoclonal antibodies, TGFβ ligand traps, antisense oligonucleotides, and vaccine-based approaches) has been reported by pre-clinical and clinical studies to reverse radioresistance of irradiated cells and boost the immune system against cancer (Formenti et al., 2018; Farhood et al., 2020; Chen et al., 2021; Kim et al., 2021; Liu et al., 2021). The present study identified SMAD3 as an additional key player in the TGFβ-related molecular cascade (Millet and Zhang, 2007) that determines the response to nCRT. It could be hypothesized that high SMAD3 expression enhances the activation of TGFβ-related genes with proliferative, anti-apoptotic, and immune suppressive effects, globally increasing the risk of not responding to nCRT. Hence, SMAD3 could be a further druggable target and SMAD3 blocking by pharmacological strategies could represent an additional promising approach to improve the tumor radiosensitivity. Preliminary data supporting the effectiveness of targeting SMAD3 for enhancing the response to radiotherapy have been published (Akhurst and Hata, 2012; Rodriguez-Ruiz et al., 2019). However, the usefulness of those pharmacological approaches should be better investigated through appropriate clinical trials prior to enter into clinical practice.

In conclusion, the significant role of SMAD3 in identifying LARC patients who are at higher risk of not responding to nCRT treatment may be critical to improve treatment strategies. SMAD3 status in LARC at diagnosis could be considered for integration into the already known clinical risk algorithms to identify patients at high risk of poor response to the combination of chemotherapy with radiotherapy. Those patients might therefore be selected for alternative personalized neoadjuvant treatments including currently available schemes, as TNT, and with intensification of preoperative chemotherapy.

## Data Availability

The datasets presented in this article are not readily available because it is currently in use for additional analyses. Requests to access the datasets should be directed to the corresponding author.

## Abbreviations

5-FU, 5-fluorouracil; IHC, immunohistochemistry; LARC, locally advanced rectal cancer; nCRT, neoadjuvant chemoradiotherapy; OS, overall survival; PFS, progression-free survival; p-SMAD3, phosphorylated SMAD3; SMAD3, SMAD family member 3; TGF-β, transforming growth factor-β; TNT, total neoadjuvant therapy; TRG, tumor regression grade; UTR, 5′-untranslated.
