# Supplementary material for: SMAD3 Host and Tumor Profiling to Identify Locally Advanced Rectal Cancer Patients at High Risk of Poor Response to Neoadjuvant Chemoradiotherapy
Source: Front Pharmacol. 2021 Dec 24;12:778781. doi: 10.3389/fphar.2021.778781 (PMC8740633; doi:10.3389/fphar.2021.778781)
Supplement: Supplementary file 1 [file Table1.DOCX]

Supplementary Material

**Supplementary Table 1A:** *In silico* predicted functional effect of *SMAD3* rs35874463, rs1065080, rs117185005, and rs1061427 polymorphisms by Ensembl’s Variant Effect Predictor (VEP) Ensembl release 103, February 2021 and RegulomeDB v2.0. Only the most relevant data are showed in the Table.

| **rs ID** | **Impact** | **ClinVar Clinical Significance** | **CADD** | **GERP** | **Missense Variant prediction** | | | | | | **Regulatory Consequence** | **Splicing effect prediction** | | **RegulomeDB** | | |
| --- | --- | --- | --- | --- | --- | --- | --- | --- | --- | --- | --- | --- | --- | --- | --- | --- |
|  |  |  |  |  | **SIFT** | **PolyPhen** | **PROVEAN** | **REVEL** | **MetaLR** | **Mutation Assessor** |  | **MaxEntScan**  **(Alt/Diff/Ref)** | **dbscSNV ADA/ RT score** | **Rank Score** | **Probability score** | **Peaks** |
| rs1065080 | Modifier | benign | 18.83  likely benign | -0.98 | -- | -- | -- | -- | -- | -- | TF binding site (TEAD4, MAX)  Less like consensus sequence  CTCF binding site |  |  | 4 | 0.60906 | 80 |
| rs35874463 | Moderate | benign/likely benign | 21.1  likely benign | 2.59 | 0.5  tolerated | 0  benign | 0.14390  neutral | 0.302  likely benign | 0.287  tolerated | 0.202  Low | -- |  |  | 2b | 0.60537 | 17 |
| rs117185005 | Low | benign/likely benign | 4.018  likely benign | -5.18 | -- | -- | -- | -- | -- | -- | CTCF binding site  Splice region variant | 5.527/2.355/7.882  High potential to diminish splicing | 0.00148/0.036 | 4 | 0.60906 | 48 |
| rs1061427 | Modifier | benign | 17.05  likely benign | -0.29 | -- | -- | -- | -- | -- | -- | -- | -- | -- | 4 | 0.60906 | 184 |

**Abbreviations**: chr, chromosomal; TF, transcriptional factors; UTR, untranslated region

**Supplementary Table 1B:** *In silico* predicted functional effect of *SMAD3* rs745103, rs744910 and rs17228212 intronic polymorphisms by HaploReg v.4.1, Ensembl’s Variant Effect Predictor (VEP) Ensembl release 103, February 2021 and RegulomeDB v2.0. Only the most relevant data for each genetic variant are reported in the Table.

| **General data from Haploreg and Ensembl’s VEP** | | | | **HaploReg^&^** | | | | | | | | **Ensembl’s VEP** | | | | **RegulomeDB** | | |
| --- | --- | --- | --- | --- | --- | --- | --- | --- | --- | --- | --- | --- | --- | --- | --- | --- | --- | --- |
| **dbSNP ID** | **Chromosome Location (GRCh38)** | **SNP Location** | **Impact** | **Promoter histone marks** | **Enhancer histone marks** | **DNAse** | **Proteins Bound** | **Motifs changed** | **NHGRI/EBI GWAS** | **GRASP QTL** | **Selected eQTL** | **CADD** | **GERP** | **Associated Phenotypes** | **PubMed (PMID)** | **Rank^** | **Score^^** | **Peaks** |
| rs745103 | Chr15:67142737 | intronic | Modifier | 3 tissues  (FAT, LNG, MUS) | 21 tissues  (ESC, ESDR, LNG, IPSC, FAT, STRM, BRST, BLD, MUS, SKIN, BRN, GI, ADRL, HRT, PLCNT, OVRY, PANC, CRVX, LIV, VAS, BONE) | 16 tissues (ESDR,ESDR,ESDR,LNG,SKIN,SKIN,HRT,LNG,MUS,LNG,MUS,MUS,VAS,BRN,SKIN,SKIN) | 1 hit (GR) | 4 altered motifs (Foxc1,MIF-1,Nrf-2,Pou3f1) |  |  |  | 0.780  likely benign | -3.18 | yes | 7 item | 4 | 0.60906 | 37 |
| rs744910* | Chr15:67154447 | intronic | Modifier |  | 10 tissues  (ESC, LNG, FAT, BRST, BLD, STRM, SKIN, PANC, MUS, LIV) | 7 tissues  (ESDR,BLD,PLCNT,MUS,BLD,SKIN,LNG) | 1 hit (MAX) | 2 altered motifs (GR,Ik-3) | 1 hit |  | 1 hit | 5.153  likely benign | 0.22 | yes | 17 item | 3a | 0.85505 | 19 |
| rs17228212** | Chr15:67166301 | intronic | Modifier | 2 tissues  (ESC, IPSC) | 15 tissues  (ESC, ESDR, LNG, IPSC, FAT, BRST, MUS, BRN, SKIN, VAS, GI, ADRL, KID, OVRY, SPLN) | 6 tissues  (ESC,BLD,SKIN,MUS,VAS,SKIN) |  | 2 altered motifs (FXR,GCNF) | 1 hit | 3 hits |  | 4.384  likely benign | -3.02 | yes | 37 item | 2a | 0.47489 | 8 |

*rs744910: the rs744910 haploblock includes also the rs10152544 (r^2^=0.98) and rs11634793 (r^2^=1)

** rs17228212: the rs744910 haploblock includes also the rs17227883 (r^2^=0.96), rs11630297 (r^2^=0.96), rs10152307 (r^2^=0.96), rs731874 (r^2^=0.96), rs72187533 (r^2^=0.97), rs10152987 (r^2^=0.97), rs35738694 (r^2^=0.99), rs11852653 (r^2^=0.99), rs72743481 (r^2^=0.93) and rs11634560 (r^2^=0.91)

^ Rank score ranges from 1 to 7 with the lower value indicating the stronger evidence for a variant to be in a functional region. 4= TF binding + DNase peak; 3a= TF binding + any motif + DNase peak; 2a= TF binding + matched TF motif + matched DNase Footprint + DNase peak

^^ Probability score ranges from 0 to 1, with 1 being most likely to be a regulatory variant.

Abbreviation: LD, linkage disequilibrium; SNP, single nucleotide polymorphism; CADD, Combined Annotation Dependent Depletion.
