# Supplementary figures and images for: SMAD3 Host and Tumor Profiling to Identify Locally Advanced Rectal Cancer Patients at High Risk of Poor Response to Neoadjuvant Chemoradiotherapy
Source: Front Pharmacol. 2021 Dec 24;12:778781. doi: 10.3389/fphar.2021.778781 (PMC8740633; doi:10.3389/fphar.2021.778781)

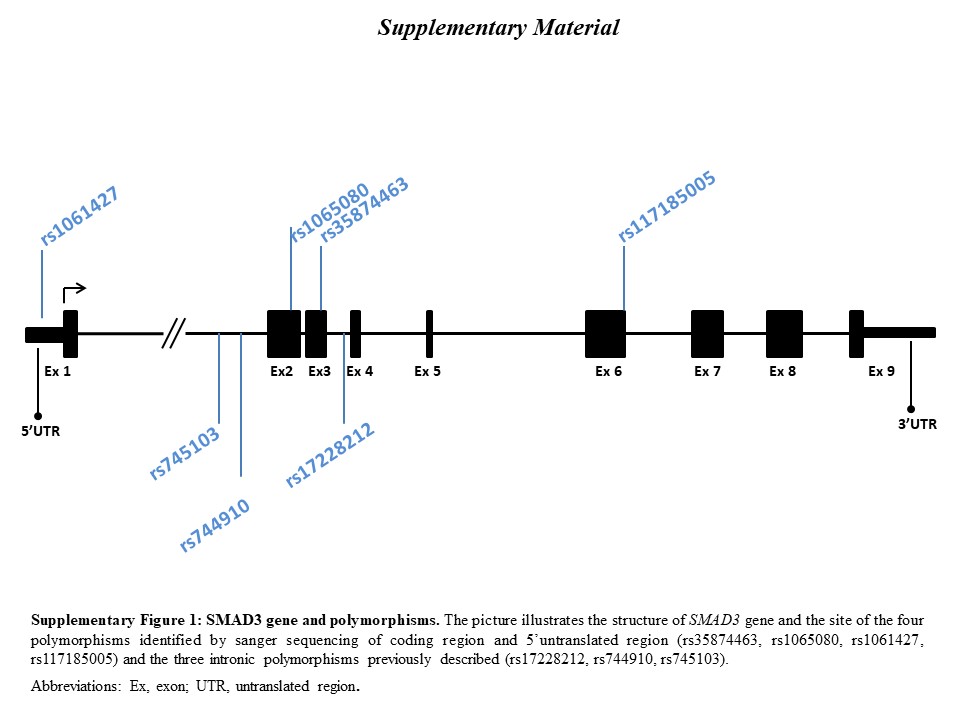

Supplement: Supplementary file 2 [file Image1.jpg]
